# Facilitation of non-nociceptive bladder activity and suppression of somatic afferent inhibition by hypogastric afferents in cats

**DOI:** 10.3389/fnins.2025.1555152

**Published:** 2025-02-20

**Authors:** Bing Shen, Jicheng Wang, Changfeng Tai, Jonathan M. Beckel

**Affiliations:** ^1^Department of Urology, University of Pittsburgh, Pittsburgh, PA, United States; ^2^Department of Pharmacology and Chemical Biology, University of Pittsburgh, Pittsburgh, PA, United States; ^3^Department of Bioengineering, University of Pittsburgh, Pittsburgh, PA, United States

**Keywords:** overactive bladder (OAB), neuromodulation, hypogastric nerve, tibial nerve, pudendal nerve

## Abstract

**Introduction:**

While it is well known that the sensory arm of the micturition reflex is mediated by A-δ afferents in the pelvic nerve, the urinary bladder is also innervated by afferents in the hypogastric nerve (HGN), whose role in micturition is less well understood. We have recently determined that stimulation of HGN afferents can facilitate nociceptive bladder activity in the cat induced by intravesical instillation of acetic acid. The aim of the present study, then, was to determine if activation of HGN afferents could similarly facilitate normal bladder activity in the cat, evoked by saline distension. Additional experiments examined if HGN stimulation could modulate the inhibitory effects of tibial or pudendal neuromodulation on bladder capacity.

**Methods:**

Continuous infusion cystometry was performed in 9 (4 male, 5 female) α-chloralose anesthetized cats in which the HGNs were transected bilaterally. Bipolar electrodes were used to stimulate the central end of the cut HGNs at varying intensities (1-15 V) and frequencies (1-40 Hz) during bladder filling with saline (1-2 ml/min) until a micturition contraction occurred. Tripolar cuff electrodes were also placed on the left pudendal and left tibial nerve to examine the effect of neuromodulation on bladder capacity during cystometry.

**Results:**

Hypogastric nerve stimulation (HGNS) at 30 Hz and 15 V significantly reduced bladder capacity to 80.01 ± 3.86% of control. Tibial or pudendal nerve stimulation (5 Hz, 2 T) significantly increased bladder capacity to 187.6 ± 20.2% or 193.8 ± 19.0% of control, respectively. Simultaneous HGNS significantly reduced the efficacy of tibial nerve neuromodulation (reduced to 158.7 ± 18.7% of control) and but only partially suppressed the bladder inhibition induced by pudendal neuromodulation (reduced to 161.9 ± 18.0% of control).

**Conclusion:**

These results suggest that HGN afferents (possibly C-fiber nociceptors) from the bladder or other pelvic organs may facilitate normal bladder activity to cause bladder overactivity. Additionally, HGN afferents may influence the efficacy of tibial or pudendal neuromodulation therapy in treating OAB.

## Introduction

It has been known for some time that the micturition reflex is mediated by non-nociceptive Aδ-fiber afferents in the pelvic nerve (Fowler et al., 2008; de Groat, 2006). On the other hand, C-fiber bladder afferents in the pelvic nerve do not respond to physiological bladder distension (Habler et al., 1990), but nociceptive bladder stimuli such as irritation/infection can sensitize these silent pelvic C-fibers to bladder distension, leading to bladder overactivity and/or pain (Fowler et al., 2008; de Groat, 2006; Habler et al., 1990; Cervero, 1994; Ness and Gebhart, 1990).

In addition to the pelvic nerve, the bladder is also innervated by the hypogastric nerve (HGN). However, the function of HGN afferents in bladder control (Cervero, 1994; Ness and Gebhart, 1990; McMahon et al., 1995) is relatively unknown. Previous animal studies (Xu and Gebhart, 2008; Moss et al., 1997; Bahns et al., 1986; Floyd et al., 1976; Guo et al., 2020) have shown that HGN afferent activity increases as intravesical pressure increases, indicating the existence of non-nociceptive, mechanosensitive, HGN afferent Aδ-fibers that innervate the bladder. However, bilateral transection of the HGNs (Mitsui et al., 2001; Cruz and Downie, 2006; Dmitrieva et al., 2001; Rudy et al., 1991) has no effect on normal reflex bladder activity, suggesting that non-nociceptive HGN afferents might not be involved in controlling physiological bladder activity like the Aδ-fibers in the pelvic nerve. Therefore, the function of HGN afferents from the bladder is currently uncertain. Our recent study (Guo et al., 2020) has determined that the HGN contains subtypes of bladder afferent fibers similar to those found in the pelvic nerve: (1) low-threshold mechanosensitive fibers that respond in a graded fashion to bladder distension, (2) high-threshold mechanosensitive that only respond to noxious distension, and (3) “silent” nociceptive fibers, which are mechanically insensitive but can be sensitized to respond to bladder distension by intravesical instillation of acetic acid. Physiologically, stimulation of the HGN in cats at intensities strong enough to activate C-fibers can facilitate nociceptive bladder overactivity induced by acetic acid irritation (Zhang et al., 2019), indicating a role for nociceptive HGN C-fiber afferents in bladder overactivity and nociception. The goal of this study is to further determine the possible role of HGN afferents in control of non-nociceptive bladder activity induced by bladder distention. Additionally, we were interested in how any modulatory effect of HGN afferent stimulation on bladder capacity could affect tibial or pudendal neuromodulation of bladder activity which might influence treatment options for bladder pathology.

## Methods

### Surgical procedures

The Animal Care and Use Committee of the University of Pittsburgh approved all protocols involving the use of animals in this study. The surgical procedures carried out for these experiments are identical to our previously published study (Zhang et al., 2019). A total of nine adult cats (four males and five females, weight: 3.3–5.7 kg) were used in this study. The animals were initially anesthetized with isoflurane (2–5% in oxygen) during surgery. Then, a catheter was inserted into the left cephalic vein and anesthesia was switched to α-chloralose (65 mg/kg initial dose and supplemented as needed throughout the experiment). The right carotid artery was also catheterized to monitor systemic blood pressure, and a tracheotomy tube was implanted to maintain airway patency. A pulse oximeter (9,847 V; NONIN Medical, Plymouth, MN) attached to the tongue was used to monitor heart rate and blood oxygen levels.

Following a midline laparotomy, the ureters were isolated, tied, cut, and drained externally. Then the proximal urethra was exposed, and a double lumen catheter was inserted into the bladder via a small incision. One lumen of the catheter was used to infuse the bladder with saline at a rate of 1–2 mL/min, while the other lumen was used to record intravesical pressure via a connection to a pressure transducer. The HGNs were transected bilaterally, their central ends tied together and attached to bipolar stainless-steel hook electrode to deliver electrical stimulation. To prevent the abdominal cavity from drying out, and to keep the electrodes electrically isolated, the abdominal skin flaps were retracted and sutured to allow the abdominal cavity to be filled with warm (35–37°C) mineral oil. In eight animals, a tripolar cuff electrode (NC223pt, MicroProbe, Gaithersburg, MD) was also implanted on the left pudendal nerve and the left tibial nerve for neuromodulation of the bladder activity.

### Experimental protocol

**Figure 1:**
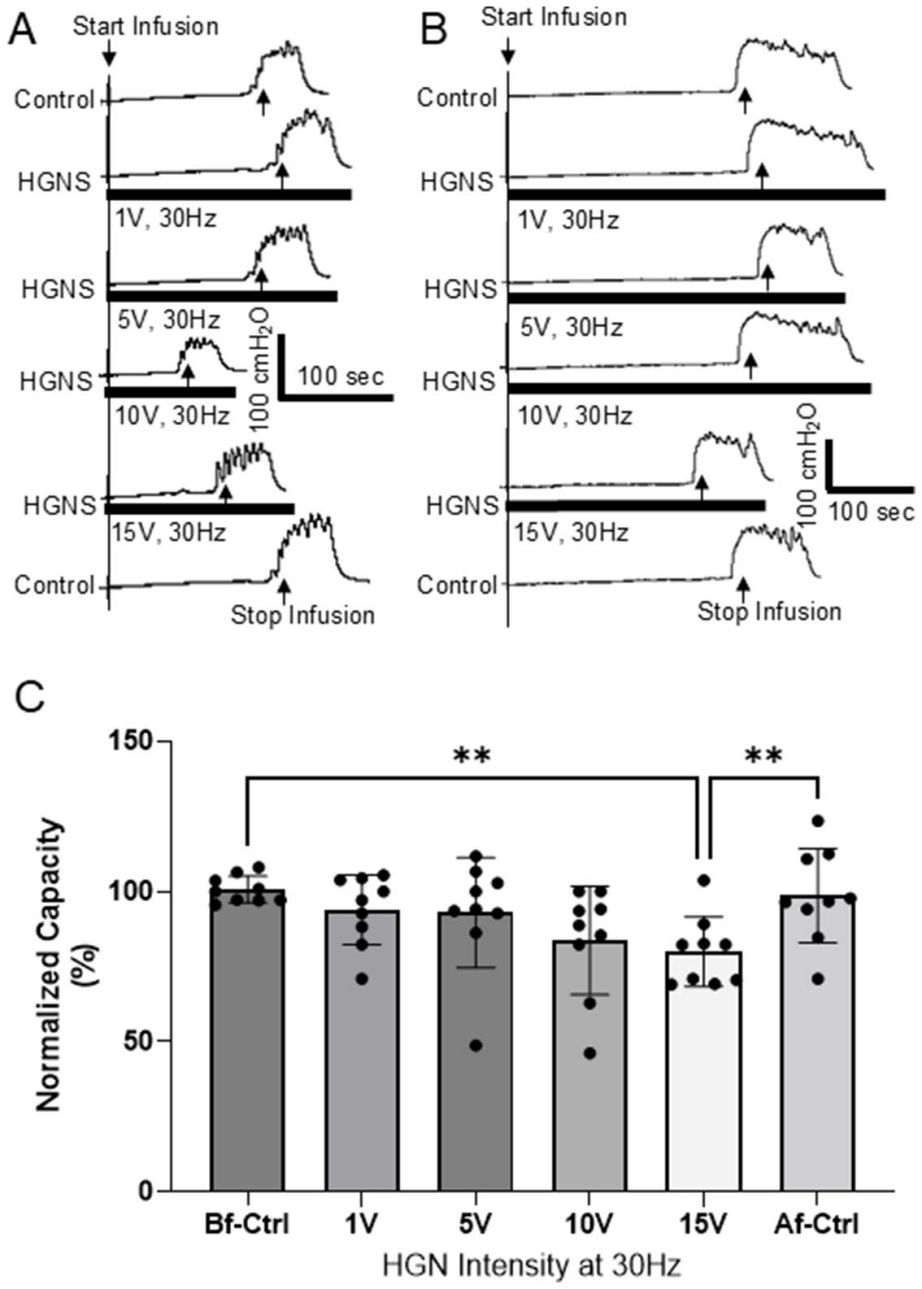
Hypogastric nerve stimulation (HGNS) facilitates micturition reflex by reducing bladder capacity. **(A)** Repeated cystometrograms (CMGs) showing that HGNS (30 Hz, 0.2 ms) at 10 V is optimal to reduce bladder capacity. **(B)** HGNS at 15 V is optimal to reduce bladder capacity in a separate animal. Black bar under bladder pressure trace indicates the stimulation duration. Infusion rate = 2 mL/min. **(C)** Bladder capacity is significantly (*p* = 0.0009, ANOVA) reduced by 30 Hz HNS at 10–15 V. *N* = 9 cats.

At the beginning of each experiment, multiple (3–5) cystometrograms (CMGs) were performed by infusing the bladder with saline (1–2 mL/min) to determine the bladder capacity, which was defined as the volume threshold to induce a bladder contraction of large amplitude (>30 cmH_2_O) and long duration (>20 s). Once the control bladder capacity was determined, 30 Hz HGN stimulation (HGNS) at intensities of 1, 5, 10, or 15 V was tested in sequence during repeated saline CMGs to determine if it could facilitate the micturition reflex by reducing the bladder capacity (*N* = 9 cats). After testing the different intensities, two control CMGs without stimulation were performed. Then, the most effective HGNS intensity (either 10 V or 15 V, see Figure 1) was selected to further determine the effect of different HGNS frequencies (1–40 Hz) during repeated saline CMGs (*N* = 9 cats). The HGNS frequencies were tested in a random order. At the end of the frequency test, two additional control CMGs without stimulation were performed.

After the most effective HGNS frequency (30 Hz) and intensity (10 or 15 V) in each animal was determined, the intensity threshold (T) for tibial nerve stimulation (TNS, 5 Hz, 0.2 ms) or pudendal nerve stimulation (PNS, 5 Hz, 0.2 ms) to induce toe twitch or anal twitch, respectively, was determined. Then, TNS or PNS (5 Hz at 2 T intensity) was applied during a CMG to inhibit micturition reflex and increase bladder capacity. Following TNS/PNS CMG, the effective HGNS (30 Hz, 10 or 15 V) was applied simultaneously with the TNS/PNS during another CMG to determine the effect of HGNS on TNS/PNS inhibition of bladder activity. After determining the effect of HGNS on TNS/PNS inhibition, two control CMGs without stimulation were performed. The TNS and PNS were tested in a random order. After each CMG, the bladder was emptied, and a 5-min waiting period was observed before starting the next CMG.

### Data analysis

Bladder capacity measured during each CMG was normalized to the control CMG in each group of experiments. All data are presented as means ± SEM. Repeated measures ANOVA (with Geisser–Greenhouse correction) followed by Tukey’s multiple comparison post-test was used to detect statistical significance (*p* < 0.05). GraphPad Prism 10.4 was used for statistical analysis and figure preparation.

## Results

### Effect of HGNS intensity and frequency on micturition reflex

**Figure 2:**
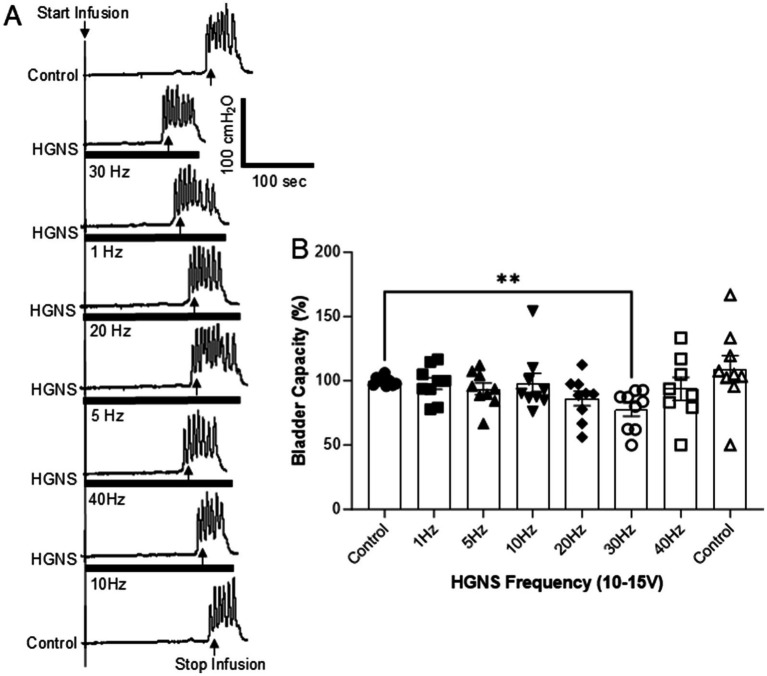
Frequency-dependent facilitation of micturition reflex by hypogastric nerve stimulation (HGNS). **(A)** Repeated cystometrograms (CMGs) showing that HGNS (15 V, 0.2 ms) at 30 Hz is most effective in reducing bladder capacity. Black bar under bladder pressure trace indicates the stimulation duration. Infusion rate = 1 mL/min. **(B)** Bladder capacity is significantly (*p* = 0.0051, ANOVA) reduced by HGNS (10–15 V, 0.2 ms) at 30 Hz. *N* = 9 cats.

HGNS facilitates the micturition reflex by significantly reducing the bladder capacity measured during saline CMGs (Figures 1, 2). This facilitation is sensitive to HGNS intensity (Figure 1) and frequency (Figure 2). At 30 Hz, HGNS is not effective at intensities below 10 V. HGNS significantly reduced bladder capacity (80.01 ± 3.86%) at an intensity of 15 V (Figure 1A), when all animals were analyzed as a group. However, it became clear during the course of our experiments that the animals could be separated into two groups: (1) those that demonstrated a reduced bladder capacity only at 10 V (Figure 1A, *N* = 4 cats) or (2) those that required 15 V (Figure 1B, *N* = 5 cats). In those animals that responded at 10 V, bladder capacity returned to control at 15 V. Regarding frequency of stimulation, 30 Hz was the most effective, other frequencies did not reduce bladder capacity (Figure 2). There were no observed differences in effective frequency or intensity observed between male and female cats.

### Effect of HGNS on tibial or pudendal inhibition of micturition reflex

**Figure 3:**
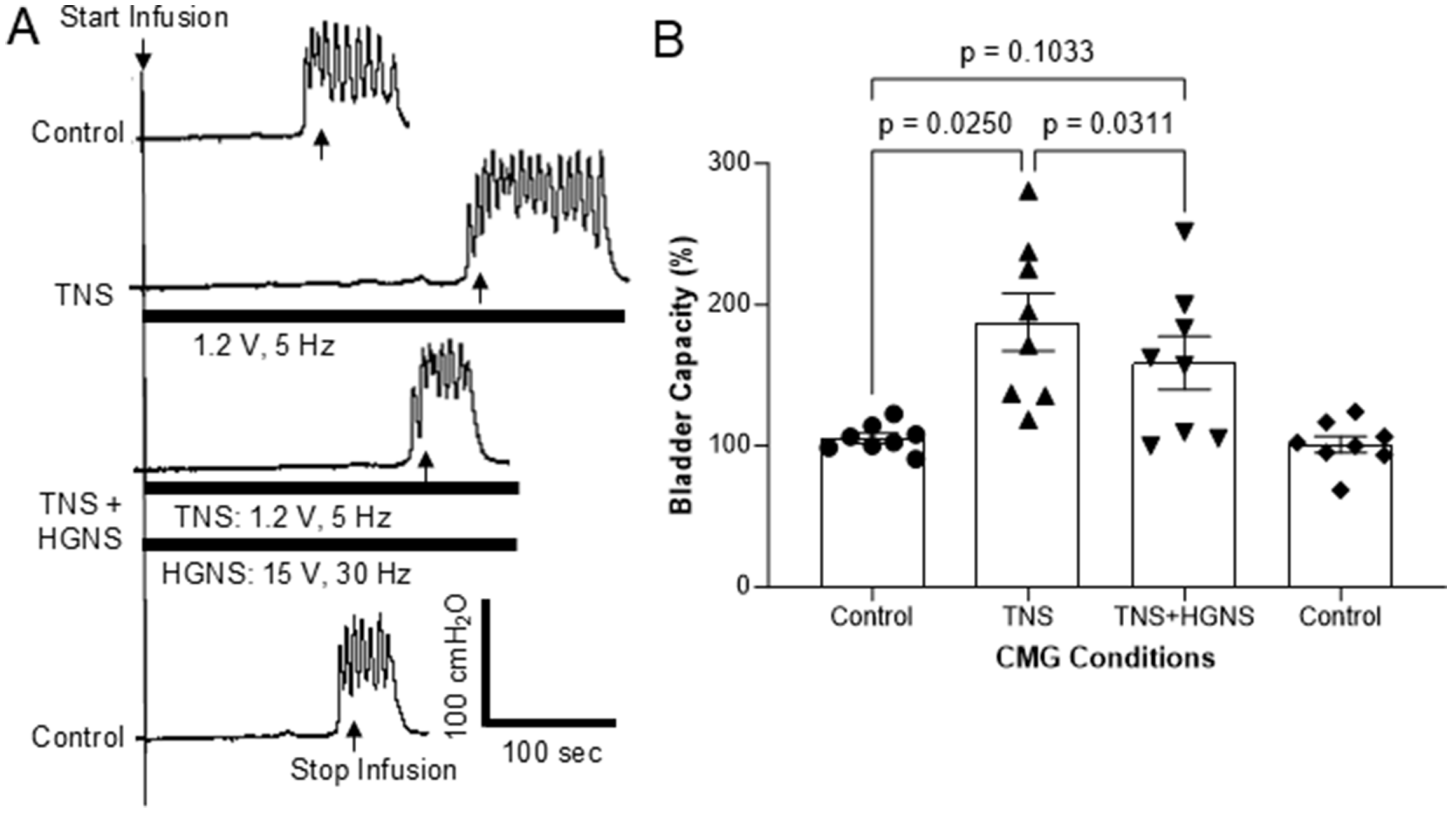
Hypogastric nerve stimulation (HGNS) suppresses bladder inhibition induced by tibial nerve stimulation (TNS). **(A)** Repeated cystometrograms (CMGs) showing that HGNS (15 V, 30 Hz, 0.2 ms) reduces the large bladder capacity produced by TNS (1.2 V, 5 Hz, 0.2 ms). Black bar under bladder pressure trace indicates the stimulation duration. Infusion rate = 1 mL/min. The threshold intensity for TNS to induce toe twitch is 0.6 V. **(B)** Bladder capacity is significantly (*p* = 0.0250, ANOVA) increased by TNS, but it is significantly (*p* = 0.0311, ANOVA) reduced by HGNS (10–15 V, 30 Hz, 0.2 ms). *N* = 8 cats.

Tibial nerve stimulation (TNS, 5 Hz at 2 T intensity) significantly (*p* = 0.0250) increased bladder capacity to 187.6 ± 20.2% of control (Figures 3A,B). HGNS (30 Hz, at the most effective intensity for the animal) applied simultaneously with TNS significantly (*p* = 0.0311) reduced the large bladder capacity to 158.7 ± 18.7% of control (Figures 3A,B). When compared to unstimulated controls, bladder capacity during simultaneous TNS/HGNS was no longer statistically different (*p* = 0.1033, Figure 3B). Subsequent unstimulated CMGs exhibited similar bladder capacity as control CMGs that occurred prior to TNS/HGN stimulation (Figures 3A,B).

**Figure 4:**
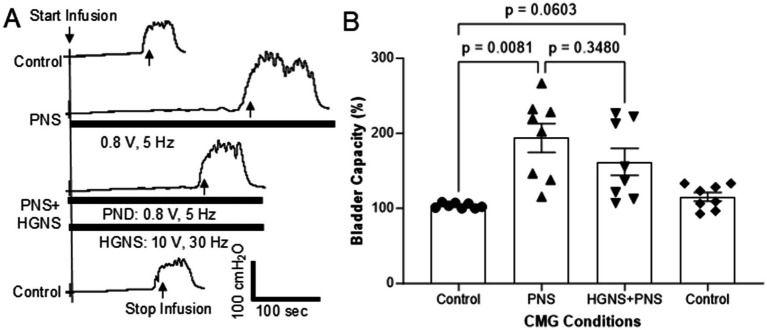
Effect of hypogastric nerve stimulation (HGNS) on bladder inhibition induced by pudendal nerve stimulation (PNS). **(A)** Repeated cystometrograms (CMGs) showing that HGNS (10 V, 30 Hz, 0.2 ms) reduces the large bladder capacity produced by PNS (0.8 V, 5 Hz, 0.2 ms). Black bar under bladder pressure trace indicates the stimulation duration. Infusion rate = 1.5 mL/min. The threshold intensity for PNS to induce anal twitch is 0.4 V. **(B)** Bladder capacity is significantly (*p* = 0.0081, ANOVA) increased by PNS, but it is not significantly (*p* = 0.0603, ANOVA) reduced by HGNS (10–15 V, 30 Hz, 0.2 ms). *N* = 8 cats.

Pudendal nerve stimulation (PNS, 5 Hz at 2 T intensity) also significantly (*p* = 0.0081) increased bladder capacity to 193.8 ± 19.0% of control (Figures 4A,B). Contrary to what we observed with TNS, HGNS applied simultaneously with PNS did not significantly (*p* = 0.3480) reduce the large bladder capacity (161.9 ± 18.0% of control, Figures 4A,B). However, when compared to unstimulated control CMGs, simultaneous PNS/HGNS bladder capacity was no longer significantly different (*p* = 0.0603). Subsequent unstimulated CMGs returned bladder capacity to normal.

## Discussion

This study in anesthetized cats shows that HGN afferents can facilitate non-nociceptive bladder activity induced by saline distention of the bladder (Figures 1, 2). This facilitation is sensitive to HGNS intensity (Figure 1) and frequency (Figure 2). Additionally, stimulation of HGN afferents can also reduce tibial afferent inhibition (Figure 3) of the micturition reflex. These results reveal a potential role of HGN afferents in modulating reflex bladder activity and in tibial/pudendal neuromodulation of micturition reflex.

HGNS is effective in facilitating micturition reflex at 10–15 V but not at 1–5 V (Figure 1). Our previous study (Zhang et al., 2019) in cats has shown that HGNS at 1–5 V activates the myelinated Aδ-fibers, while unmyelinated C-fibers require 10–15 V to activate. Therefore, in this study it is likely that the HGN facilitation of micturition reflex is due to activation of the C-fibers in HGN. Meanwhile, our study also indicates that electrical stimulation of the HGN at the intensities consistent with activation of Aδ-fibers has minimal effects on the micturition reflex. This may explain why surgical transection of the HGN has no effect on the normal micturition reflex activity (Mitsui et al., 2001; Cruz and Downie, 2006; Dmitrieva et al., 2001; Rudy et al., 1991) since HGN C-fibers are not activated by normal physiological bladder conditions. Moreover, as the HGN also contains afferents from other pelvic organs which were most likely activated by our electrical stimulation, our research also suggests that activation of HGN afferents from other pelvic organs (not from the bladder) may facilitate normal micturition reflex to cause bladder overactivity. Pathology of the pelvic organs often show co-morbidity, which is thought to occur via crosstalk between the pelvic afferents innervating the visceral organs (Ustinova et al., 2006). Our study suggests that it might also be possible for an HGN-to-pelvic crosstalk from other pelvic organs to modulate micturition or bladder sensations.

This study shows that a very narrow intensity range (10 or 15 V for different animals) is required to facilitate micturition reflex. For those cats (*N* = 4) where 10 V is effective, stronger stimulation at 15 V loses the effectiveness (Figure 1A). Meanwhile, for those cats (*N* = 5) where 15 V is effective, 10 V is not effective (Figure 1B). Because we are stimulating the HGN central to the transection, only HGN afferents are stimulated, and there is no motor response that can be used to determine the threshold (T) intensity to normalize stimulation across different animals. Therefore, we used 1, 5, 10, and 15 V since our previous study (Zhang et al., 2019) in cats showed that 10–15 V activated HGN C-fiber afferents. However, due to the different impedances between the electrode and nerve in different animals, the same group of afferents may require slightly different intensities in order to be activated. These different impedances are most likely a result of intersubject variations, such as nerve size and differences in the organization of nerve fibers within the neuron, as well as variations in experimental setup, such as variability in the electrodes used or their placement on the nerve. Nevertheless, this narrow effective intensity range indicates a very specific group of afferents in the HGN can facilitate micturition reflex. Activating more afferents of higher intensity threshold will, on the contrary, lose the facilitation (Figure 1A).

It should be noted that our previous study (Zhang et al., 2019) did not detect a facilitatory effect of HGNS on bladder capacity during saline distension but did detect a facilitatory effect during acetic acid distension. HGNS of a single, high intensity (16 V) was used in our previous study to screen different frequencies in six cats (Zhang et al., 2019), which may have resulted in ineffective results in half of the cats based on our current study, obscuring a significant result. Additionally, our previous study (Zhang et al., 2019) used 20 Hz instead of 30 Hz to screen the different intensities, which is ineffective as shown in our current study (Figure 2B). Our present study, with almost twice the animals and a more systematic approach to testing stimulation parameters, is able to observe HGNS-mediated reductions in bladder capacity.

Both tibial and pudendal neuromodulation have been used to treat patients with overactive bladders (OAB) (Bartley et al., 2013). Our study indicates that HGN afferents may play an important role in influencing the efficacy of these neuromodulation therapies. As shown in Figure 3, simultaneous HGNS and TNS significantly reduced the increase in bladder capacity induced by TNS alone. Additionally, while simultaneous HGNS did not significantly alter pudendal nerve-mediated neuromodulation, bladder capacity was decreased in multiple individual experiments within our cohort (see Figure 4B). This suggests that activation of HGN afferents by bladder or other pelvic organ pathology may reduce the efficacy of tibial/pudendal neuromodulation in treating OAB. This possibility breaks away from the traditional thinking that tibial/pudendal neuromodulation is only interacting with the pelvic afferents that control micturition function. It may also suggest a potential clinical complication for the use of TNS/PNS neuromodulation in the treatment of cases of bladder pathology that occur concomitantly with other pelvic organ pathologies, such as IBS.

In summary, this study in anesthetized cats reveals that HGN afferents can facilitate non-nociceptive bladder activity and suppress bladder inhibition induced by tibial or pudendal neuromodulation. These results imply a role for HGN afferents, possibly nociceptive C-fibers based on the stimulation intensities required to activate them, in bladder overactivity and in bladder inhibition by tibial/pudendal neuromodulation.

## Data Availability

The raw data supporting the conclusions of this article will be made available by the authors, without undue reservation.
